# Investigation of High-Temperature Normal Infrared Spectral Emissivity of ZrO_2_ Thermal Barrier Coating Artefacts by the Modified Integrated Blackbody Method

**DOI:** 10.3390/ma15010235

**Published:** 2021-12-29

**Authors:** Tong Zhang, Xuyao Song, Gongjin Qi, Baolin An, Wei Dong, Yan Zhao, Zhiyong Wang, Xiaosu Yi, Zundong Yuan, Yunlong Zhao, Luge Sun, Hongyu Mao

**Affiliations:** 1 Department of Materials Science and Engineering, Beihang University, Beijing 100191, China; toryman@126.com (T.Z.); jennyzhaoyan@buaa.edu.cn (Y.Z.); 2 Beijing Institute of Aeronautical Materials, Beijing 100195, China; zywang910@163.com (Z.W.); yi_xiaosu@sina.com (X.Y.); 3 Beijing Aeronautical Technology Research Center, Beijing 102213, China; qgjin@sina.com (G.Q.); maohongyu474@163.com (H.M.); 4 National Institute of Metrology (NIM), Beijing 100029, China; anbl@nim.ac.cn (B.A.); yuanzd@nim.ac.cn (Z.Y.); nimzhaoyl@126.com (Y.Z.); sunluge98@126.com (L.S.); 5 Department of Composites, University of Nottingham Ningbo China, Ningbo 315048, China; 6 Department of Energy and Environment, Beijing University of Civil Engineering and Architecture (BUCEA), Beijing 100191, China

**Keywords:** normal infrared spectral emissivity, integrated blackbody method, ZrO_2_ coating artefact, thermal barrier coating, uncertainty, high-temperature

## Abstract

Zirconium oxide (ZrO_2_) is widely used as the thermal barrier coating in turbines and engines. Accurate emissivity measurement of ZrO_2_ coating at high temperatures, especially above 1000 °C, plays a vital role in thermal modelling and radiation thermometry. However, it is an extremely challenging enterprise, and very few high temperature emissivity results with rigorously estimated uncertainties have been published to date. The key issue for accurately measuring the high temperature emissivity is maintaining a hot surface without reflection from the hot environment, and avoiding passive or active oxidation of material, which will modify the emissivity. In this paper, a novel modified integrated blackbody method is reported to measure the high temperature normal spectral emissivity of ZrO_2_ coating in the temperature range 1000 °C to 1200 °C and spectral range 8 μm to 14 μm. The results and the associated uncertainty of the measurement were estimated and a relative standard uncertainty better than 7% (*k* = 2) is achieved.

## 1. Introduction

Thermal barrier coating materials capable of withstanding extreme temperatures are continually being developed for the next generation of gas turbines. The internationally recognized method for measuring the normal infrared spectral emissivity of coating materials under a high-temperature condition is the direct measurement based on the definition of emissivity, namely, via the normal radiance ratio between the surface of the measured sample and that of the reference blackbody [1]. However, the main problems can be concluded as high temperature sample heating without cavity effect suppression and accurate measurement of sample surface temperature.

Previous emissivity measurement results reported by some major national measurement research institutions, such as the National Institute of Standards and Technology (NIST) [2], the National Physical Laboratory (NPL) [3], the NASA Langley Research Center [4], the Laboratoire Commun de Métrologie (LNE-Cnam) [5], the Physikalisch-Technische Bundesanstalt (PTB) [6], the National Institute of Metrology (NIM) [7], were mainly focused on the mid-temperature range, i.e., below 1000 °C. Researchers at the Nanjing University of Aeronautics and Astronautics (NUAA) [8] and the Harbin Institute of Technology (HIT) [9] have tried to increase the temperature range up to 1500 °C, but have mainly focused on measurement schemes and device construction rather than the uncertainty evaluation. All the studies above obtained the radiance ratio by measuring the radiation signal of samples and blackbodies separately. The high-temperature sample heating methods adopt the one-dimensional back heating method or heat pipe heating method. The former method can easily cause a temperature gradient inside the sample, leading to a poor uniformity of the sample surface temperature. In the latter method, one needs to consider the influence of the cavity effect formed by coupling between the heat pipe and the sample coupling. Another difficulty of the above studies lies in the measurement of the true temperatures of the sample surface, which is usually estimated directly by radiation temperature measurement or contact temperature measurement. The former requires accurate prediction of the emissivity of the sample, while the latter one can only be used to measure the temperature at a certain depth behind the sample. In this case, the true temperature of the sample surface can only be calculated if one knows the thermal conductivity and thermal diffusivity of the material. Uncertainties in these parameters (emissivity, thermal conductivity and diffusivity) contribute to the temperature measurement uncertainty. More importantly, these contributions rise at higher temperature, becoming the primary cause for the measurement uncertainty.

In this paper, a novel high-temperature infrared spectral emissivity measurement method is introduced, namely a modified integrated blackbody method. The classical integral blackbody principle was first proposed in 1986 by Vader et al. [10]. It applies to the case where samples under certain emissivity conditions are inserted into a cavity with a large depth to diameter ratio, such that the cavity formed by the sample surface coupling with the cavity wall can be considered as an effective blackbody, i.e., with an emissivity close to 1. In 1994, Postlethwait et al. [11] from Pennsylvania State University (PSU) proposed the high temperature spectral emissivity measurement method using this principle for the first time and set up an emissivity measurement device using the vertical free falling sample method for the temperature range of 500 °C to 1000 °C. From 2004 to 2010, Herdrich et al. [12,13,14] from the Institut für Raumfahrtsysteme (IRS), University of Stuttgart, designed and built an emissivity measurement device for thermal protection materials using the integrated blackbody method and adopted a narrow-band thermometer as the radiance measurement device for the study of plasma wind motion.

However, the integrated blackbody method still needs to be improved. First of all, there is little discussion about the two main error sources: the effective emissivity of the formed integrated blackbody and the neglecting of the temperature drop during the integral blackbody process, especially for the second error source. Secondly, the behavior of other non-ideal factors has not been studied in detail, such as the size-of-source effect (SSE) of the infrared optical system and the linearity of the FTIR radiation detection system spectral responsivity.

Therefore, in this study, we have improved the classical integrated blackbody method by treating both error sources, and analyzed other factors such as the size-of-source effect (SSE) of the infrared optical system and the linearity of the spectral responsivity of the FTIR radiation detection system. The details were reported in Section 3.

The facility used to study emissivity was a Fourier transform infrared spectrometer based infrared emissivity measurement system. The system can cover the spectral range of 1 μm to 14 μm, and the temperature range of 1000 °C to 1500 °C. The ZrO_2_ coating showed semi-transparent in the near- and short- wavelength infrared region, and opaque in the long wavelength infrared region [15,16,17,18]. In this preliminary study, we reported first results for measurements of emissivity in the wavelength range 8 μm to 14 μm. Together with rigorous estimates of uncertainty. The relative expanded uncertainty is less than 7% (*k* = 2) over the whole wavelength range.

## 2. Materials and Methods

### 2.1. Raw Materials

ZrO_2_ powders (99%) with an average size of 100 nm were obtained from Shanghai Macklin Biochemical Co., Ltd., Shanghai, China. CeO_2_ powders with an average size of 80 nm were analytical reagent grade and purchased from Sinopharm Chemical Reagent Co., Ltd., Beijing, China. The main properties of these powders were listed in Table 1.

### 2.2. Preparation of ZrO_2_ Coating

**Figure 1:**
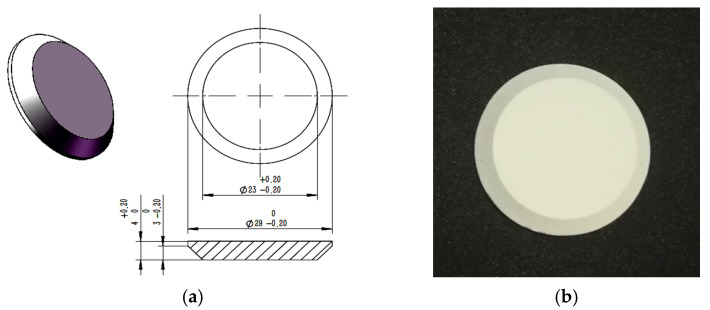
The ZrO_2_ coating artefact sample and its dimensions: (**a**). The dimensions of the substrate; (**b**). The photo of ZrO_2_ coating artefact sample.

The dimensions of the ZrO_2_ coating artefacts and the substrate are shown in Figure 1, and the sample was prepared at BIAM. The ZrO_2_ based coatings are formed on Ni-based super-alloy substrate (the operating temperature upper limit 1300 °C). The substrate is a truncated cone. The diameter of the side with coating artefacts is 23 mm, and the backside of the substrate is 29 mm. The thickness of the coating artefact is approximately 60 μm.

The ZrO_2_ based coating artefact samples were prepared by ball-milling and heat treatment. The ball-milling treatment process of ZrO_2_ based samples is as following: first, 200 g of raw ZrO_2_ powders, 20 g of raw CeO_2_ powders, 600 g of zirconia grinding ball, 450 mL of ethanol, and 28 g of stearic acid are added to a ball mill tanks. Second, they are fixed to the planetary ball-mill (QM-3SP4) and the ball milling rate is set to be 500 rpm for ball milling time of 5 h. Third, the mixed powders are washed with deionized water and ethanol to remove fully the impurities on the surface of the powders and then dried under vacuum and oven drying conditions. Finally, the ball-milled mixed powders are calcined in muffle furnace in air at 1400 °C for 2 h with heating rate of 5 °C/min. After being crushed and ground, the sieved sample was mixed with home-made silicate adhesive (60 wt% in the mixture), followed by adjusting the viscosity to 24 s by adding some DI-water. The ZrO_2_ based coating was deposited on the surface of Ni-based super-alloy substrate using a compressed air spraying method with a spray pressure of 0.4 MPa. The coated sample was settled in the air at room temperature for 12 h, baked in a drying oven at 100 °C for 30 min, and then calcined in muffle furnace at 1000 °C for 30 min to obtain the final ZrO_2_ coated artefact. The roughness of ZrO_2_ coating was 3.2 μm to 4.8 μm.

### 2.3. Material Characterizations

**Figure 2:**
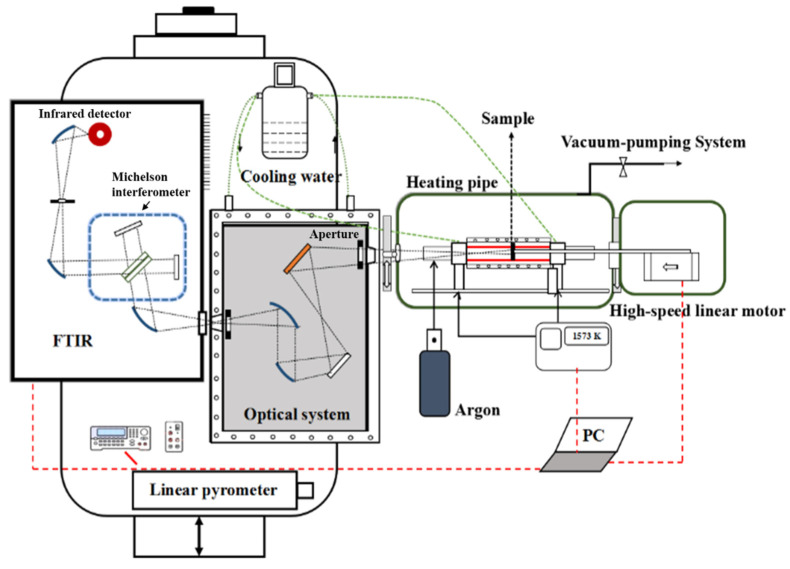
The schematic diagram of the BIAM and NIM infrared spectral emissivity measurement facility.

The scheme of the high-temperature infrared spectral emissivity measurement facility is shown in Figure 2. The main components are the radiation source sub-system using a high-temperature blackbody furnace for heating the integrated blackbody and sample; the infrared radiation detection sub-system uses the FTIR with the external reflective infrared optical system (a main gold-plated concave mirror radius of curvature 500 mm, two gold-plated off-axis ellipsoidal sub-mirrors both with 76.2 mm focal length, and a 2 mm diameter field stop with 300 mm distance from the cavity opening); the sample surface temperature drop measurement sub-system uses a standard linear photoelectric reference radiation thermometer (LP4) (traced to the NIM radiation temperature reference standard, the highest level standard available in China) at the wavelength 0.65 μm with 750 mm focal length. The dimensions of sighting areas of FTIR and LP4 are both 2 mm.

The thermogauge furnace uses a low-voltage, high current source (full load voltage 24 V and full load current 2000 A) to allow rapid heating from room temperature to 3000 °C, namely a heating rate of 3 °C/s, with a temperature control precision ±0.1 °C through the low voltage and high current. The heated part of the radiation source sub-system is the cylindrical graphite cavity (inner diameter 38 mm, total length 360 mm) The furnace is protected by argon with a 2 atm pressure. The sample fixed in a graphite crucible (the sample surface diameter 23 mm) is located in the center of the graphite cavity, that is, 180 mm away from the cavity opening. A contact thermometer is a S-type thermocouple of 3 mm diameter protected in a ceramic tube of 5 mm diameter. It is located at the cavity bottom and in contact with the back of the sample, providing a nominal temperature for the integrated blackbody. The ZrO_2_ coated artefact surface coupling with the graphite cavity forms an integrated blackbody cavity. We have measured the temperature control stability of the sample surface at the temperature 1000 °C over 30 min to be better than 0.5 °C by experimental measurement. To push the sample quickly from the center of the cavity to the front, the rear surface of the sample crucible is attached to a graphite rod, the other end of which is connected to a high-speed linear motor. The positioning accuracy of the high-speed linear motor is 0.01 mm, and the time to move the sample from center to opening is 147 ms.

The FTIR (Bruker Vertex 70 with an uncooled deuterated tri-glycine sulfate (DTGS) pyro-electric detector of area 1 mm^2^, the wavelength range 1–20 μm) applies the rapid scan model with the scan frequency of 60 kHz. double-sided, double-forward motion spectrum sweeping mode and synchronous trigger scheme of the moving mirror Michelson interferometer are adopted to achieve the transient measurement of spectral radiance, yielding more than 10 output spectrums within 150 ms.

The optical system is protected by a water-cooled environmental radiation shield to avoid both stray light and the thermal effects of the high temperature radiation on the optical components.

## 3. Results

### 3.1. Principle

**Figure 3:**
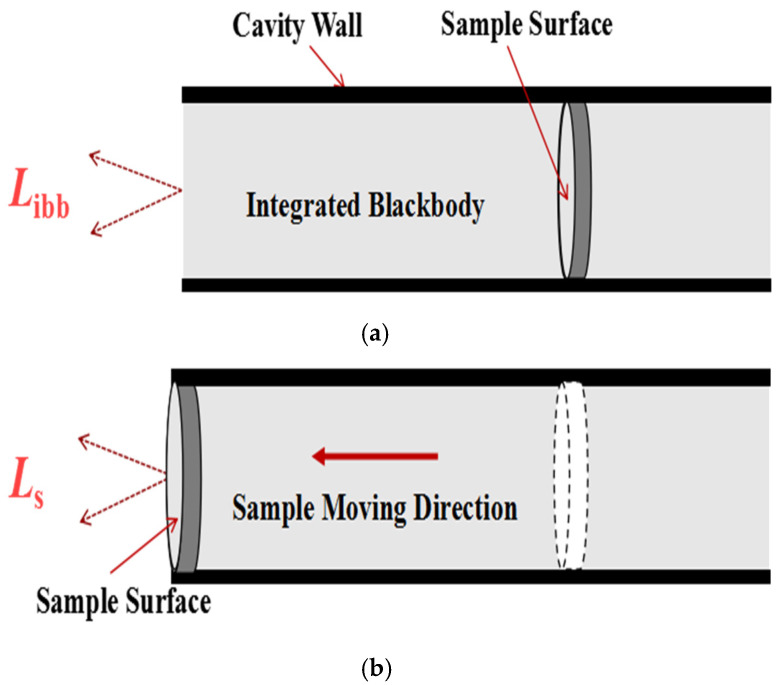
The schematic of the emissivity measurement by means of the integrated blackbody method: (**a**). The integrated blackbody formed by the sample surface on the cavity bottom coupled with the cavity wall; (**b**). The sample being moved from the bottom to the opening of the cavity.

When a sample is located at the bottom of the cavity, it is couples with the side wall of the high emissivity blackbody cavity to form an integrated blackbody with an emissivity of approximately 1, as shown in Figure 3a. The spectral radiance *L_ibb_* of the integrated blackbody can be given by:

(1) $$L_{ibb}(\lambda ,T_{ibb})=k_{ibb}\varepsilon_{ibb}(\lambda ,T_{ibb})L_{Pla}(\lambda ,T_{ibb})+L_{env}(\lambda ,T_{env})$$

where *λ* is the wavelength, *T_ibb_* is the temperature of the integrated blackbody, *k_ibb_* is the observation factor when measuring the integrated blackbody radiance, *ε_ibb_* is the spectral emissivity of the integrated blackbody, *L_Pla_* is the ideal blackbody spectral radiance determined by Planck’s law, *L_env_* is the environment background radiance, and *T_env_* is the environment background temperature.

When the sample is located at the cavity opening, as shown in Figure 3b, its surface spectral radiance *L_s_* can be given by:

(2) $$L_{s}(\lambda ,T_{s})=k_{s}\varepsilon_{s}(\lambda ,T_{s})L_{Pla}(\lambda ,T_{s})+L_{env}(\lambda ,T_{env})$$

where *T_s_* is the true temperature of the sample surface, *k_s_* the observation factor when measuring the sample surface radiance, *ε_s_* the spectral emissivity of the sample surface. The observation factors *k_ibb_* and *k_s_* are related to the optical system [19].

The spectral emissivity of the sample surface can be obtained based on the Equations (1) and (2), and given by:

(3) $$\varepsilon_{s}(\lambda ,T_{s})=\varepsilon_{ibb}(\lambda ,T_{ibb})\cdot \frac{k_{ibb}}{k_{s}}\cdot \frac{L_{s}(\lambda ,T_{s})-L_{env}(\lambda ,T_{env})}{L_{ibb}(\lambda ,T_{ibb})-L_{env}(\lambda ,T_{env})}\cdot \frac{L_{Pla}(\lambda ,T_{ibb})}{L_{Pla}(\lambda ,T_{s})}$$

The spectral output *S* of the FTIR is a dimensionless value, which can be obtained by direct output of FTIR. It is defined by the spectral radiance *L*, and given by [20]:

(4) $$S(\lambda ,T)=R(\lambda ,T)L(\lambda ,T)+R(\lambda ,T_{env})L_{env}(\lambda ,T_{env})$$

where *R* is the spectral responsivity. Above 1000 °C, the environment background spectral radiance *L_env_* is several orders of magnitude lower than the spectral radiance of the integrated blackbody *L_ibb_*, so can be ignored.

According to the Equations (3) and (4), the sample surface spectral emissivity is given by:

(5) $$\varepsilon_{s}(\lambda ,T_{s})=\varepsilon_{ibb}(\lambda ,T_{ibb})\cdot \frac{k_{ibb}}{k_{s}}\cdot \frac{R(\lambda ,T_{ibb})S_{s}(\lambda ,T_{s})}{R(\lambda ,T_{s})S_{ibb}(\lambda ,T_{ibb})}\cdot \frac{L_{Pla}(\lambda ,T_{ibb})}{L_{Pla}(\lambda ,T_{s})}$$

where the ratio *k_ibb_/k_s_* can be evaluated by the size-of-source effect (SSE) [15], *R*(*λ*,*T_ibb_*)/*R*(*λ*,*T_s_*) evaluated by the linearity of spectral responsivity of the FTIR detection system [21], and *L_Pla_*(*λ*,*T_ibb_*)/*L_Pla_*(*λ*,*T_s_*) based on the Planck–Wien approximation at high-temperature expressed as [22]:

(6) $$\frac{L_{Pla}(\lambda ,T_{ibb})}{L_{Pla}(\lambda ,T_{s})}=\exp[-\frac{c_{2}}{\lambda }(\frac{1}{T_{ibb}}-\frac{1}{T_{s}})]$$

where *c*_2_ is the second radiation constant.

One can calculate the true sample surface temperature *T_s_* by measuring the temperature drop Δ*T* of the sample when it is moved from the bottom to the opening of the cavity, that is *T_s_* = *T_ibb_* − Δ*T*. Thus, this effectively avoid the difficult problem of measuring the true temperature of the sample surface in the high-temperature emissivity measurement method. Equation (6) can be re-written expressed as:

(7) $$\frac{L_{Pla}(\lambda ,T_{ibb})}{L_{Pla}(\lambda ,T_{s})}=\exp[-\frac{c_{2}}{\lambda }(\frac{1}{T_{ibb}}-\frac{1}{T_{s}})]=\exp[-\frac{c_{2}}{\lambda }(\frac{1}{T_{ibb}}-\frac{1}{T_{ibb}-\Delta T})]=C(\lambda ,T_{ibb},\Delta T)$$

where *C* is defined as the sample surface temperature drop correction factor.

Therefore, the sample surface infrared spectral emissivity by means of the integrated blackbody is given by:

(8) $$\begin{array}{cc} \varepsilon_{s}(\lambda ,T_{ibb},\Delta T,T_{env}) & =\varepsilon_{ibb}(\lambda ,T_{ibb})\cdot \frac{k_{ibb}}{k_{s}}\cdot \frac{R(\lambda ,T_{ibb})S_{s}(\lambda ,T_{s})}{R(\lambda ,T_{s})S_{ibb}(\lambda ,T_{ibb})}\cdot \exp[-\frac{c_{2}}{\lambda }(\frac{1}{T_{ibb}}-\frac{1}{T_{ibb}-\Delta T})]\\ & =\varepsilon_{ibb}(\lambda ,T_{ibb})\cdot \frac{k_{ibb}}{k_{s}}\cdot \frac{R(\lambda ,T_{ibb})S_{s}(\lambda ,T_{s})}{R(\lambda ,T_{s})S_{ibb}(\lambda ,T_{ibb})}\cdot C(\lambda ,T_{ibb},\Delta T) \end{array}$$

### 3.2. Measurement Results of the Infrared Spectral Emissivity of ZrO_2_ Coating Artefact

**Figure 4:**
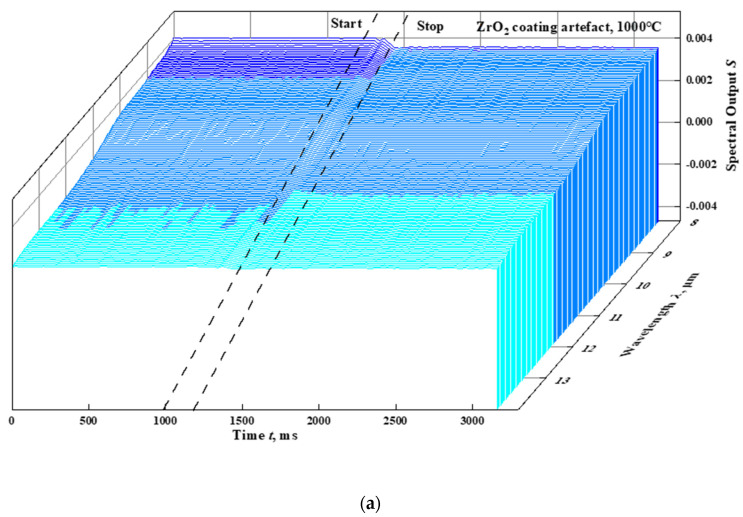

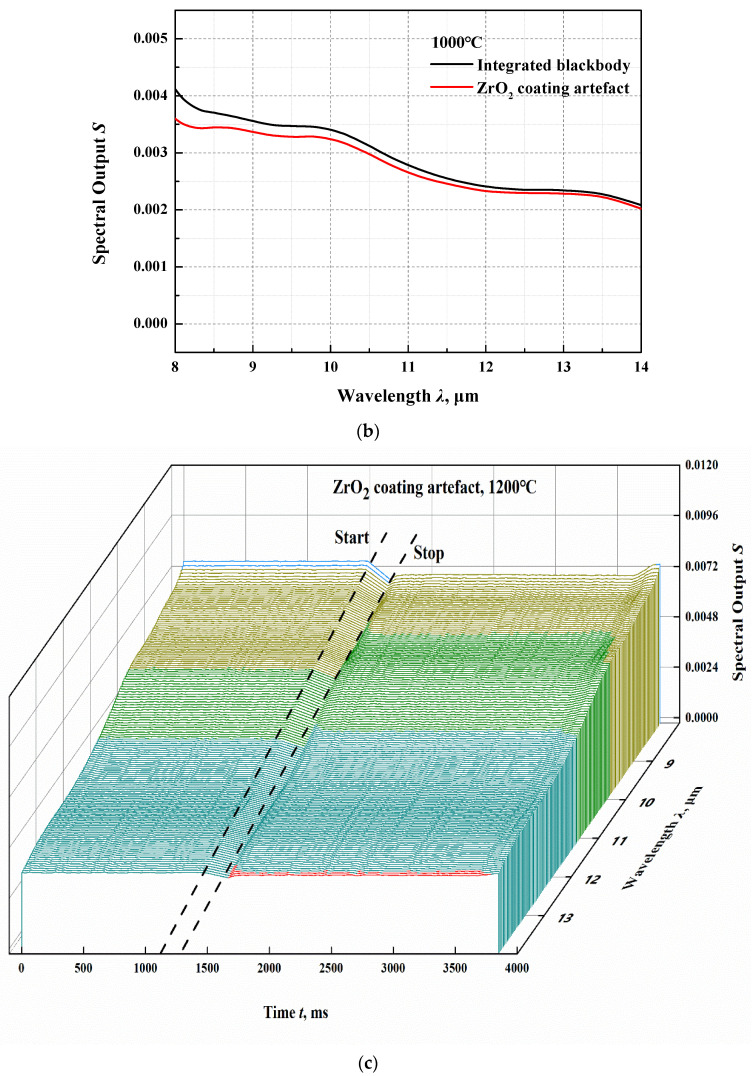

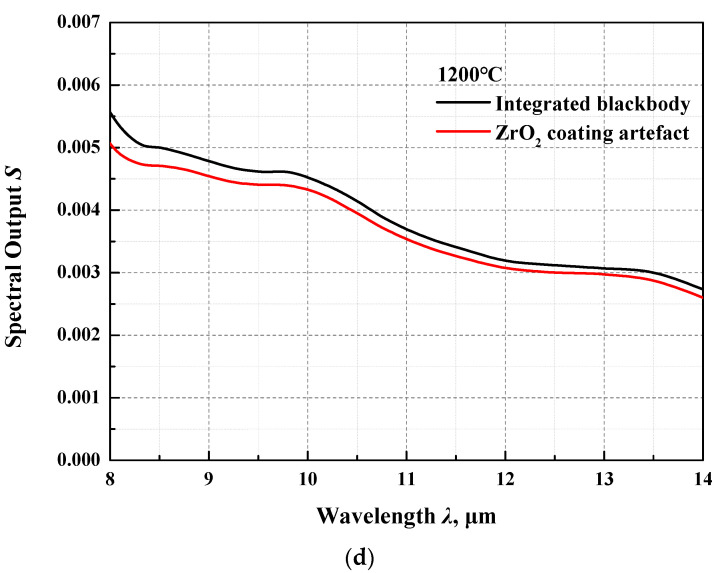
The FTIR spectral outputs of the infrared spectral emissivity measurement of ZrO_2_ coating artefacts at the temperatures 1000 °C and 1200 °C. (**a**). The FTIR spectral outputs of the ZrO_2_ coating artefact in the whole measurement process at the temperature 1000 °C. (**b**). The effective FTIR spectral outputs of the integrated blackbody and the ZrO_2_ coating artefact at the temperature 1000 °C. (**c**). The FTIR spectral outputs of the ZrO_2_ coating artefact over the whole measurement process at the temperature 1200 °C. (**d**). The effective FTIR spectral outputs of the integrated blackbody and the ZrO_2_ coated artefact at the temperature 1200 °C.

**Figure 5:**
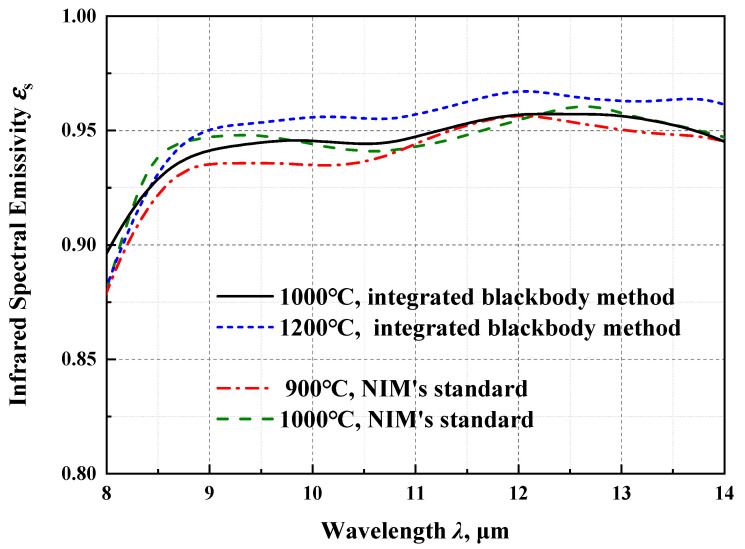
The measurement and NIM’s verification results of the infrared spectral emissivity of the ZrO_2_ coating artefacts.

The emissivity of the ZrO_2_ coated artefacts in the wavelength range of 8–14 μm are measured at nominal integrated blackbody temperatures of 1000 °C and 1200 °C. The FTIR spectral outputs for the whole measurement processes are shown in Figure 4a,c, respectively. Just before the artefact is pushed, the FTIR spectral outputs for the integrated blackbody are collected. Once the artefact has reached the cavity opening and is stopped, the FTIR spectral outputs from the ZrO_2_ coated artefact surface are recorded. The effective FTIR spectral outputs for the integrated blackbody and the artefacts moving towards the cavity opening at the temperatures of 1000 °C and 1200 °C are shown in Figure 4b,d. The average of three measurements of the spectral emissivity are shown in Figure 5, where the repeatability is larger than 98.5%. The experimental verifications of emissivity measurement results are carried out at temperatures of 900 °C and 1000 °C, based on the NIM standard for the material’s infrared spectral emissivity measurement in the temperature range of 50–1000 °C (the highest standard level of China [7]) by means of the direct measurement method. According to the comparisons, the emissivity deviations varies are less than 0.02 at 1000 °C in the wavelength range of 8–14 μm, and the good consistency shows the effectiveness of the modified integrated blackbody method.

## 4. Discussion

### 4.1. The Integrated Blackbody Effective Emissivity

We obtain the effective emissivity of the integrated blackbody by numerical simulation using the Monte-Carlo ray tracing method which is the recognized by the international metrology community [22]. In this situation, the expression for the effective emissivity *ε_ibb_* of the integrated blackbody is given by [23]:

(9) $$\varepsilon_{ibb}(\lambda )=1-\frac{1}{N}\sum_{i=1}^{N}\sum_{k=1}^{M}\rho {\left(\lambda \right)}^{k}$$

where *N* is the total number of ray tracing, *M* the number of reflections, *k* in the trace of the *i*-th branch tracing and *ρ* the reflectivity of the cavity wall. Introducing the non-isothermal boundary conditions on the high-temperature, the numerical simulation expression of the effective emissivity *ε_ibb_*, non-iso of the non-isothermal integrated blackbody is given by [24]:

(10) $$\varepsilon_{ibb,non-iso}=\frac{\varepsilon^{k}(\lambda )}{N}\exp(-\frac{c_{2}}{\lambda T_{ref}})\;\sum_{i=1}^{N}\sum_{k=1}^{M}\rho^{k-1}\exp(-\frac{c_{2}}{\lambda T_{k}})$$

where *ε* is the cavity wall emissivity, *T_ref_* the reference temperature of the integrated blackbody.

**Figure 6:**
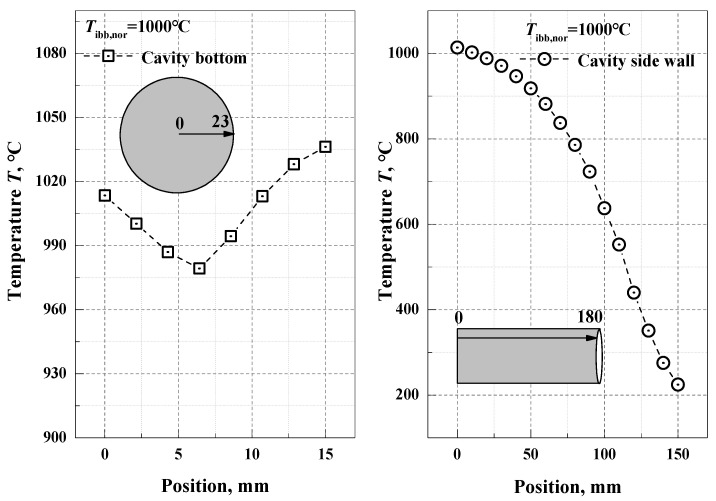
The temperature distribution of the integrated blackbody formed by ZrO_2_ coating artefact and the graphite cavity wall at the nominal temperature 1000 °C.

The numerical simulation result of the non-isothermal integrated blackbody cavity formed by ZrO_2_ coating artefact and the graphite cavity wall (*ε_Gra_* = 0.9) [25] is given for a nominal temperature of 1000 °C with the integrated blackbody taken as an example. The LP4 reference radiation thermometer is used to scan and measure the distribution of the radiation temperatures on the cavity bottom and wall surfaces at the wavelength of 0.65 μm. The results are shown in Figure 6. The true surface temperatures of the cavity bottom and cavity wall are inverted according to the estimated emissivities of the cavity bottom and cylindrical wall, which are substituted into Equation (10) to obtain the numerical simulation results. According to the temperature field measurement results for the integrated blackbody, the simulated emissivity of the integrated blackbody is 0.956 in the opaque wavelength range, and 0.993 in the wavelength range of (8–14) μm.

### 4.2. Surface Temperature Drop of ZrO_2_ Coated Artefact

Although the temperature drop when the sample was pushing from the cavity center to opening has been ignored in the literatures [11,12], this temperature drop was measured and analyzed in this paper and this will help to improve the accuracy of measurement results. The process of pushing the sample pushed from the cavity center to the opening can be discretized into n rings of equal area, and the boundary layer at the cavity opening can be considered as a room temperature blackbody. Using the radiation heat transfer theory of thermodynamics, the radiation heat transfer between the sample surface at any discrete ring position and the surface of the cavity body can be obtained [26]. For the two states when pushing the sample out from the furnace bottom to the opening and the sample was steady at the furnace opening, the forced convective and natural convective heat transfer models were used, respectively. When the sample was pushing from the furnace bottom to the opening with an initial temperature of 1000 °C, the Reynolds number was obtained from the sample average velocity, sample size and gas thermo-physical properties. Then, the convective heat transfer coefficient was obtained according to the single tube forced convection heat transfer formula. The qualitative temperature for gas thermo-physical properties was selected as the average temperature of furnace bottom and furnace mouth wall. When the sample was steady at the furnace opening with an initial temperature of 1000 °C, the natural convection heat transfer coefficient was obtained according to sample size and gas thermo-physical properties, where the qualitative temperature for gas thermo-physical properties was selected as the average temperature of the sample temperature and room temperature. The results showed that for the ZrO_2_ samples, the effect of convective heat transfer on temperature is roughly 0.1% order magnitude at 1000 °C. Therefore, the convection heat loss can be neglected.

Assuming thermal stability in the cavity and neglecting the influence of convection, one can calculate the heat transfer flow Φ*_i_* using difference between the output radiation and the input radiation, which is given by:

(11) $$\Phi_{i}=A_{rad}\varepsilon_{s}\sigma T_{s}^{4}-\sum_{i=2}^{n}A_{i}F_{i-1}J_{i}=A_{rad}(\varepsilon_{s}\sigma T_{s}^{4}-\sum_{i=2}^{n}F_{1-i}J_{i})$$

where *A_rad_* is the heat transfer area, *ε_s_* the emissivity of the sample surface, *σ* the Stefan-Boltzmann radiation constant, *F* the angular coefficient, and *J* the effective radiation energy.

I heat transfer flow Φ*_i_* can be expressed as:

(12) $$\Phi_{i}=\frac{dQ_{i}}{dt}=mC_{pi}\frac{dT_{i}}{dt}$$

where *Q* is the amount of heat, *t* the conduction time, *M* the mass, and *C_p_* is the heat capacity at constant pressure. Thus, the sample surface temperature drop Δ*T* across any rIng *i* is given by:

(13) $$\Delta T_{i}=\frac{dT_{i}}{dt}\Delta t_{i}=\frac{A_{rad}(\varepsilon_{s}\sigma T_{si}^{4}-\sum_{i=2}^{n}F_{1-i}J_{i})}{mC_{pi}T_{si}}\Delta t_{i}$$

Therefore, the total sample surface temperature drop when the sample is pushed from the cavity center to the opening is given by:

(14) $$\Delta T_{\mathrm{total}}=\sum_{i}^{n}\Delta T_{i}$$

When it is assumed that the number of discrete rings is only 1, the sample surface carries out only the radiation heat exchange with the room temperature blackbody. Equation (14) can be expressed as:

(15) $$\Delta T_{l}=\frac{dT}{dt_{l}}t_{l}=\frac{A_{rad}\varepsilon_{s}\sigma T^{4}}{mC_{p}(T)}t_{l}$$

where *t*_l_ is the sample push-out time from the cavity center to the opening. Thus, the amount of the heat exchange when the sample surface is exposed to the cavity opening must be greater than the value when the sample is displaced inside the cavity. The sample surface temperature drop Δ*T*_l_ in Equation (15) can be considered as the maximum theoretical temperature drop. The true temperature drop of the sample surface must lie within the temperature interval (0 − Δ*T*_l_). In addition, the heat exchange state of the sample surface within a short time after reaching the cavity opening can be considered to be approximately the same as the value of the sample surface just reaching the cavity opening, that is, the sample surface always exchanges heat with the room temperature environment. Thus, according to the Equation (15), the maximum temperature drop of sample surface can be equal to the slope of the temperature change on the sample surface measured after reaching the cavity opening over the time and the sample pushed-out time. The maximum temperature drop Δ*T*_max_ can be given by:

(16) $$\Delta T_{\max}=\frac{dT_{\mathrm{mea}}}{dt_{\mathrm{mea}}}t_{l}$$

where *T*_mea_ is the measured temperature change of the sample surface, *t*_mea_ is the measurement time. The temperature change of the sample surface is measured can experimentally using a LP4 reference radiation thermometer (detection wavelength 0.65 μm) with a rapid data collection model. The measurement time of the temperature change of the sample surface after reaching the cavity opening is specified as 200 ms, that is, the temperature within 200 ms after the sample reaches the cavity opening is continuously measured. Above proposed method in this paper is used to evaluate the temperature drop of the sample surface.

**Figure 7:**
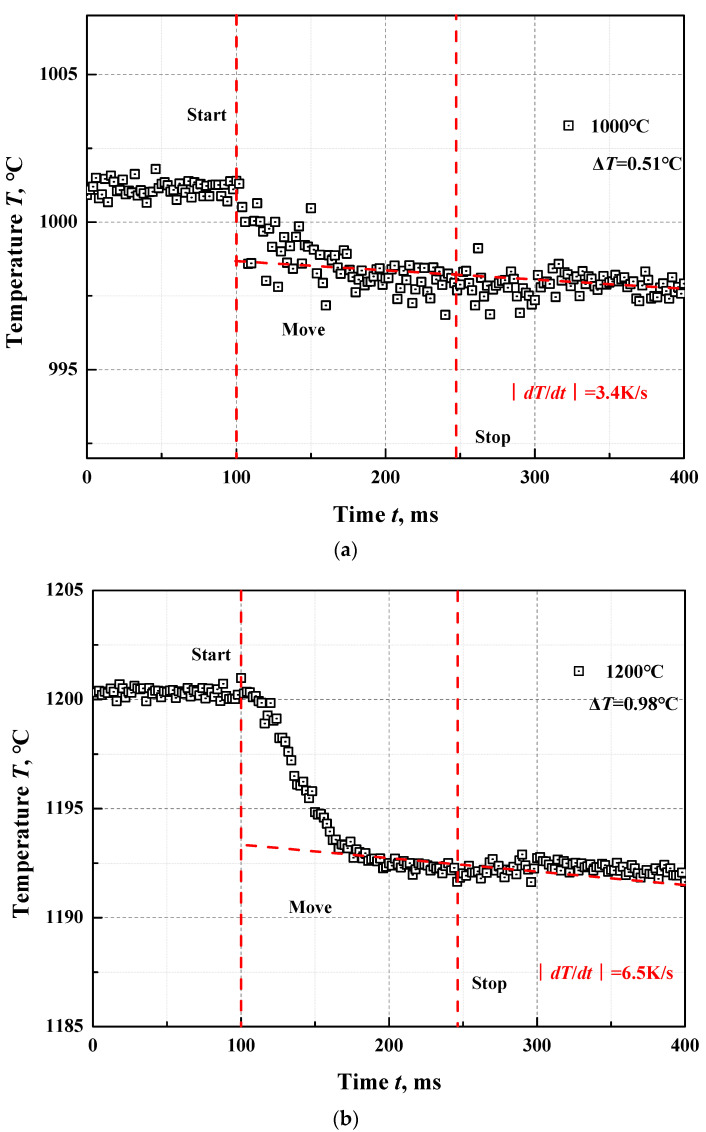
The surface temperature change measurements and the maximum temperature drops of the ZrO_2_ coating artefacts. (**a**). The surface temperature change measurement and the maximum temperature drop of the ZrO_2_ coating artefact at the temperature closed to 1000 °C. (**b**). The surface temperature change measurement and the maximum temperature drop of the ZrO_2_ coating artefact at the temperature 1200 °C.

The maximum surface temperature drops of the ZrO_2_ coating artefacts are measured using the above-mentioned evaluation method at temperatures of 1000 °C and 1200 °C, as shown in Figure 7. It should be noted that the temperature drops in Figure 7 refers to the radiation temperature drops at 0.65 μm. To accurately obtain the sample surface true temperature drop, another experiment was carried out at 1000 °C to measure the normal spectral emissivity of ZrO_2_ at 0.65 μm, with a result of 0.18. With the measured ZrO_2_ emissivity and the radiation temperature drops, the true temperature drop is obtained as 0.62 °C for 1000 °C and 1.22 °C for 1200 °C. According to the temperature drops and Equation (7), the sample surface temperature drop correction facto C for the ZrO_2_ coating artefacts are obtained, respectively, with the value of 1.009 for 1000 °C and 1.013 for 1200 °C.

To check the method for evaluating the temperature drop of sample surface the sample pushed-out time was varied. A linear fit of maximum temperature drop versus push-out time was performed from 147 ms to 1604 ms. As seen in Equation (16), when the sample push-out time is vanishingly small, that is, the sample reaches the cavity opening instantly, there is, in theory, no temperature drop of the sample surface. According to the linear fitting equation, when the sample push-out time in the equation is set to zero, the calculated temperature drop of the sample surface calculated also approaches 0 °C. This comparison validates the feasibility of the evaluation method for the temperature drop of the sample surface determined by the temperature change measurement within a short time of the sample after reaching the cavity opening. However, showing this method is more widely applicable will require further research and some refinements to theory.

### 4.3. The Size-of-Source Effect (SSE) of the Infrared Optical System

**Figure 8:**
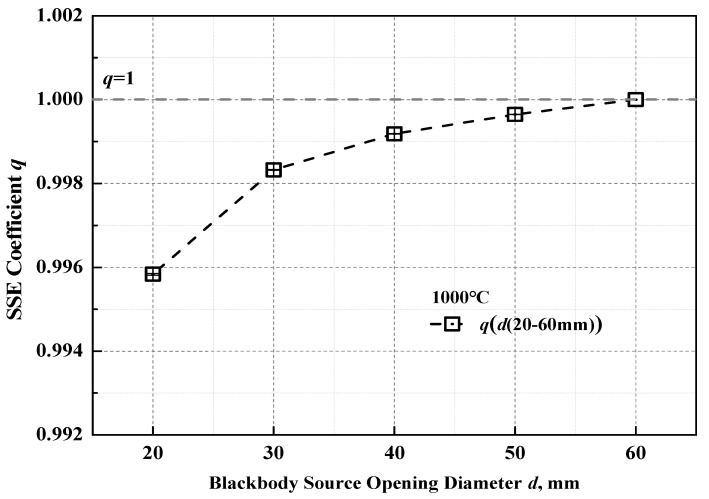
The SSE coefficient measurement results.

The size-of-source effect (SSE) arises from the effects of diffraction and scattering of target radiation, imperfection of the optical system and optical aberration. The experimental measurement of the SSE coefficient q of the reflective infrared optical system is carried out using the direct method at the temperature 1000 °C with a standard heat pipe blackbody as the radiation source (traced to the NIM variable temperature blackbody calibration facility) with a cavity opening diameter of 65 mm (temperature uniformity better than 0.6 °C) [27]. The results are shown in Figure 8.

In the actual emissivity measurement, when the sample is pushed from the integrated blackbody cavity center to the opening, the diameter of the effective measured area varies between 20 mm and 30 mm. The SSE coefficients q for the diameters of 20 mm and 30 mm are taken as the boundary conditions for the evaluation of the ratio *k_ibb_*/*k_s_* in Equation (8). We find

(17) $$\frac{k_{ibb}}{k_{s}}=\frac{q(d=30\;\mathrm{mm})}{q(d=20\;\mathrm{mm})}\approx 1.001$$

### 4.4. The Linearity of the Spectral Responsivity of the FTIR Detection System

**Figure 9:**
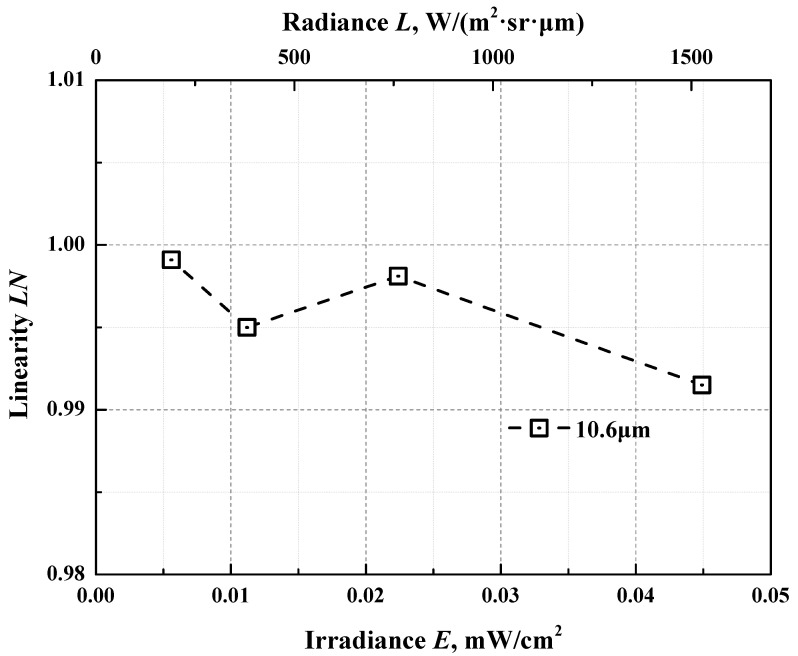
The linearity measurement results of the spectral responsivity of the FTIR radiation detection system.

If the spectral responsivity of the FTIR detection system has a linear response characteristics, the ratio *R*(*λ*,*T_ibb_*)/*R*(*λ*,*T_s_*) in Equation (8) should be equal to unity. However, due to the non-ideal characteristics of the detector and the associated amplifier circuit, that is, the spectral responsivity of the spectral detection system is nonlinear, thus *R*(*λ*,*T_ibb_*)/*R*(*λ*,*T_s_*) in Equation (8) must not be equal to 1. The linearity LN of the spectral responsivity of the FTIR radiation detection sub-system is measured using the double-aperture method of flux superposition, more details about which can be found in references [28,29]. Taken as an example, the linearity measurement result at the wavelength of 10.6 μm, which is in the middle of the wavelength range 8–14 μm is shown in Figure 9. The ratio *R*(*λ*,*T_ibb_*)/*R*(*λ*,*T_s_*) is evaluated via the corresponding linearity measurement result.

### 4.5. The Uncertainty Budgets

According to the integrated blackbody method, the measurement uncertainty of the infrared spectral emissivity of the ZrO_2_ coating artefacts mainly originates from: the integrated blackbody effective emissivity, the observation factor, the integrated blackbody-sample radiance ratio, and the sample surface temperature drop correction factor.

The model can be expressed as,

(18) $$\varepsilon_{s}(\lambda ,T_{ibb},\Delta T,T_{env})=\varepsilon_{ibb}\cdot K\cdot Ratio\cdot C$$

where,

(19) $$\varepsilon_{ibb}=\frac{\varepsilon^{k}(\lambda )}{N}\exp(-\frac{c_{2}}{\lambda T_{ref}})\;\sum_{i=1}^{N}\sum_{k=1}^{M}\rho^{k-1}\exp(-\frac{c_{2}}{\lambda T_{k}})$$

(20) $$K=\frac{k_{ibb}}{k_{s}}$$

(21) $$Ratio=\frac{R(\lambda ,T_{ibb})S_{s}(\lambda ,T_{s})}{R(\lambda ,T_{s})S_{ibb}(\lambda ,T_{ibb})}$$

(22) $$C=\exp[-\frac{c_{2}}{\lambda }(\frac{1}{T_{ibb}}-\frac{1}{T_{ibb}-\Delta T})]$$

Thus, the uncertainty transfer equation can be given as,

(23) $$u_{\varepsilon_{s}}=\sqrt{{\left(\frac{\partial \varepsilon_{s}}{\partial \varepsilon_{ibb}}u_{\varepsilon_{ibb}}\right)}^{2}+{\left(\frac{\partial \varepsilon_{s}}{\partial K}u_{K}\right)}^{2}+{\left(\frac{\partial \varepsilon_{s}}{\partial Ratio}u_{Ratio}\right)}^{2}+{\left(\frac{\partial \varepsilon_{s}}{\partial C}u_{C}\right)}^{2}}$$

The determination and evolution method of contribution components are from the uncertainty transfer method according to the emissivity measurement model. We also reference the ‘NIM’s (50–1000) °C emissivity measurement standard available in China. The uncertainty components with the associated uncertainty type are represented in Table 2, among which the type A uncertainties are taken as the standard deviation of the average of the several measurements [1], while the type B uncertainties are obtained by the verification and calibration of experimental instruments. The determinations of type B uncertainty sub-components are according to the NIM’s associated standards available in China. The combination of the contributions of the whole uncertainty components are according to the uncertainty transfer method. The relative combined standard uncertainties of the ZrO_2_ coating artefacts at both the temperatures of 1000 °C and 1200 °C are less than 3.5% (*k* = 1) and the corresponding relative expanded uncertainties are less than 7% (*k* = 2).

## 5. Conclusions

The emissivity of the ZrO_2_ thermal barrier coating artefacts were constructed at Beijing Institute of Aeronautical Materials (BIAM) and measured in the wavelength range 8–14 µm of artefacts at 1000 °C and 1200 °C at the Chinese National Institute of Metrology (NIM) using a modified integrated blackbody method home-designed and home-built facility. The main results are as follows:

(1) The effective emissivity of the integrated blackbody cavity, the temperature drop of the sample surface, the size-of-source effect SSE of the infrared optical system, and the linearity of the spectral responsivity of the FTIR radiation detection system was introduced to improve the measurement method.

(2) The method for evaluating temperature drop of the sample surface by measuring the surface temperature change has been proposed, and its feasibility is demonstrated.

(3) The ZrO_2_ coated artefacts present high emittance radiation characteristics in the long wavelength range of 8–14 μm and their infrared spectral emissivities are calculated to be near 0.95. The associated relative expanded uncertainties are less than 7% (*k* = 2) and relative combined standard uncertainty are less than 3.5% (*k* = 2).

(4) Compared with the results based on the conventional infrared spectral emissivity measurement standard at NIM, the emissivity deviation between these two is less than 0.02 at 1000 °C in the 8–14 μm. The good consistency proves the effectiveness and accuracy of the modified method proposed in this paper.

In summary, the integrated blackbody method has been improved by discussing about the two main error sources and analyzing other non-ideal factors, and new results have been obtained in this paper. In the future, further improvements can be made by adding another temperature measurement method to measure the time when the sample moved from the center of the cavity to the opening.

## Figures and Tables

**Table 1:** The properties of ZrO_2_ and CeO_2_ powders.

Properties	ZrO_2_	CeO_2_
Density	5.89 g/cm^3^	7.13 g/cm^3^
Melt point	2700 °C	2600 °C
Purity	99.99%	99.95%
Particle size	100 nm	80 nm
Crystal structure	monoclinic	cubic

**Table 2:** The uncertainty budgets for the infrared spectral emissivities of the ZrO_2_ coating artefacts at the temperatures 1000 °C and 1200 °C.

Uncertainty Components		Type	*u*_1000 °C_ (*k* = 1),%	*u*_1200 °C_ (*k* = 1),%
Integrated blackbody effective emissivity				
	Integrated blackbody temperature field	A	1.36	1.36
	Integrated blackbody temperature control stability	A	0.01	0.01
	Estimated emissivity of the ZrO_2_ coating artefact	B	0.92	0.92
	Estimated emissivity of the graphite	B	0.17	0.17
Observation factor				
	Source-size effect	A	0.01	0.01
	Distance effect	A	N. S	N. S
Integrated blackbody-sample radiance ratio				
	Spectral output repeatability	A	1.74	1.93
	Linearity of the FTIR responsivity	A	1.82	1.82
	FTIR noise	B	0.01	0.01
	FTIR wavenumber accuracy	B	0.01	0.01
	FTIR wave number repeatability	B	N. S	N. S
	Short term stability of FTIR	B	N. S	N. S
	Environmental stray radiation	A	0.24	0.21
	ThermoGauge furnace control stability	A	0.01	0.02
	ThermoGauge furnace temperature drop	A	0.31	0.38
	Sample positioning repeatability	B	N. S	N. S
Sample surface temperature drop correction factor				
	Sample surface temperature drop	A	0.35	0.67
	LP4 wavelength accuracy	B	N. S	N. S
	LP4 filter bandwidth	B	N. S	N. S
	LP4 range coefficient	B	0.02	0.03
	LP4 noise	B	0.01	0.02
	LP4 source-size effect	B	0.06	0.08
	LP4 short-term stability	B	0.11	0.13
	LP4 linearity	B	N. S	N. S
	LP4 apparent accuracy	B	N. S	N. S
Relative combined standard uncertainty *u*_c_ (*k* = 1), %			3.19	3.34
Relative expanded uncertainty *u*_c_ (*k* = 2), %			6.38	6.68

## References

[1] Hanssen L., Wilthan B., Monte C., Hollandt J., Hameury J., Filtz J.-R., Girard F., Battuello M., Ishii J. (2016). Report on the CCT supplementary comparison S1 of infrared spectral normal emittance/emissivity. Metrologia.

[2] Hanssen L., Mekhontsev S., Khromchenko V. (2004). Infrared spectral emissivity characterization facility at NIST, Thermosense XXVI. Proc. Spie.

[3] Zhang B., Redgrove J., Clark J. (2003). New apparatus for measurement of the spectral, angular, and total emissivity of solids. High Temp.-High Press..

[4] Ohlhorst C., Vaughn W., Daryabeigi K., Lewis R., Rodrigues A., Milhoan J., Koenig J. (2007). Emissivity results on high temperature coatings for refractory composite materials. Proceedings of the SPIE 17th International Expansion Symposium.

[5] Hay B., Hameury J., Fleurence N., Lacipiere P., Grelard M., Scoarnec V., Davee G. (2014). New facilities for the measurements of high-temperature thermophysical properties at LNE. Int. J. Thermophys..

[6] Krenek S., Gilbers D., Anhalt K., Taubert D.R., Hollandt J. (2015). A dynamic method to measure emissivity at high temperatures. Int. J. Thermophys..

[7] Zhang T., Dong W., Wang Z.Y., Yi X.S., Zhao Y., Yuan Z.D., Zhao Y.L. (2020). Investigation of infrared spectral emissivity of low emittance functional coating artefacts. Infrared Phys. Technol..

[8] Ren D., Tan H., Xuan Y., Han Y., Li Q. (2016). Apparatus for measuring spectral emissivity of solid materials at elevated temperatures. Int. J. Thermophys..

[9] Dai J., Wang X., Yuan G. (2005). Fourier transform spectrometer for spectral emissivity measurement in the temperature range between 60 and 1500 °C. Conference Series, Proceedings of the 7th International Symposium on Measurement Technology and Intelligent Instruments, Huddersfield, UK, 6–8 September 2005.

[10] Vader D.T., Viskanta R., Incropera F.P. (1986). Design and testing of a high-temperature emissometer for porous and particulate dielectrics. Rev. Sci. Instrum..

[11] Postlethwait M.A., Sikka K.K., Modest M.F., Hellmann J.R. (1994). High-temperature, normal spectral emittance of silicon carbide based materials. J. Thermophys. Heat Transf..

[12] Herdrich G., Löhle S., Auweter-Kurtz M., Endlich P., Fertig M., Pidan S., Schreiber E. IRS ground-testing facilities: Thermal protection system development, code validation and flight experiment development, ResearchGate. Proceedings of the 24th Aerodynamic Measurement Technology and Ground Testing Conference, AIAA-2004-2596.

[13] Schüßler M., Auweter-Kurtz M., Herdrich G., Lein S. (2009). Surface characterization of metallic and ceramic TPS-material for reusable space vehicles. Acta Astronaut..

[14] Massuti-Ballester B., Pagan A.S., Herdrich G. Determination of total and spectral emissivity of space relevant materials. Proceedings of the 8th European Symposium on Aerothermodynamics for Space Vehicles.

[15] Manara J., Arduini-Schuster M., Rätzer-Scheibe H.-J., Schulz U. (2009). Infrared-optical properties and heat transfer coefficients of semitransparent thermal barrier coatings. Surf. Coat. Technol..

[16] Manara J., Arduini-Schuster M., Keller M.H. (2011). Infrared-optical characteristics of ceramics at elevated temperatures. Infrared Phys. Technol..

[17] Manara J., Zipf M., Stark T., Arduini M., Ebert H.-P., Tutschke A., Hallam A., Hanspal J., Langley M., Hodge D. (2017). Long-wavelength infrared radiation thermometry for non-contact temperature measurements in gas turbine. Infrared Phys. Technol..

[18] Levi C.G., Hutchinson J.W., Vidal-Sétif M.-H., Johnson C.A. (2012). Environmental degradation of thermal-barrier coatings by molten deposits. MRS Bull..

[19] Ishii J., Ono A. (2001). Uncertainty estimation for emissivity measurements near room temperature with a Fourier transform spectrometer. Meas. Sci. Technol..

[20] Song X., Duanmu Q., Dong W., Li Z. (2019). Piecewise linear calibration of Fourier spectral measurement system responsivity based on the high temperature blackbody. Infrared Laser Eng..

[21] Yuan Z., Zhang J., Zhao J., Liang Y., Duan Y. (2009). Linearity study of a spectral emissivity measurement facility. Int. J. Thermophys..

[22] Zhang Z.M., Tsai B.K., Machin G. (2010). Radiometric Temperature Measurements 1. Fundamentals.

[23] Sapritsky V.I., Prokhorov A.V. (1992). Calculation of the effective emissivities of specular-diffuse cavities by the Monte-Carlo method. Metrologia.

[24] Prokhorov A.V., Hanssen L.M. (2004). Effective emissivity of a cylindrical cavity with an inclined bottom: I. Isothermal cavity. Metrologia.

[25] Song X., Dong W., Pan Y., Yuan Z., Lu X. (2021). The infrared spectral emissivity measurement of a graphite material in a high temperature range of 1000~1500 °C using integrated blackbody principle. J. Infrared Millim. Waves.

[26] Modest M.F. (2003). Radiative Heat Transfer.

[27] Hao X., Yuan Z., Lu X., Zhao W. (2011). Size-of-source effect difference between direct and indirect methods of radiation thermometers. Int. J. Thermophys..

[28] Song X., Dong W., Yuan Z., Lu X., Li Z., Duanmu Q. (2020). Investigation of the linearity of the NIM FTIR infrared spectral emissivity measurement facility by means of flux superposition method. Infrared Phys. Technol..

[29] Song X., Dong W., Yuan Z., Li X.L.Z., Qu Y., An B., Huan K. (2021). Linearity measurement of FTIR infrared spectral emissivity measurement facility determined using flux superposition at NIM. J. Phys. Conf. Ser..

